# Digital Health for Patients Undergoing Cardiac Surgery: A Systematic Review

**DOI:** 10.3390/healthcare11172411

**Published:** 2023-08-28

**Authors:** Kevin A. Wu, Sameer Kunte, Shashank Rajkumar, Vishal Venkatraman, Grace Kim, Samantha Kaplan, Syed Omar Anwar-Hashmi, Julie Doberne, Tom C. Nguyen, Shivanand P. Lad

**Affiliations:** 1 Department of Surgery, Duke University School of Medicine, Durham, NC 27710, USA; 2 Department of Neurosurgery, Yale University, New Haven, CT 06510, USA; 3 Department of Neurosurgery, Duke University Medical Center, Durham, NC 27710, USA; 4 Medical Center Library & Archives, Duke University School of Medicine, Durham, NC 27710, USA; 5 Department of Surgery, Loyola University Chicago’s Stritch School of Medicine, Maywood, IL 60153, USA; 6 Division of Cardiovascular and Thoracic Surgery, Department of Surgery, Duke University Medical Center, Durham, NC 27707, USA; 7 Division of Adult Cardiothoracic Surgery, Department of Surgery, UCSF Health, San Francisco, CA 94143, USA

**Keywords:** digital health, cardiac surgery, systematic review, mobile applications, web-based interventions, perioperative care, patient outcomes, patient engagement

## Abstract

Digital health interventions have shown promise in improving patient outcomes and experiences in various healthcare settings. However, their effectiveness in the context of cardiac surgery remains uncertain. This systematic review aims to evaluate the existing evidence on the use of digital health interventions for patients undergoing cardiac surgery. A comprehensive search of PubMed MEDLINE, Elsevier EMBASE, Elsevier Scopus databases, and ClinicalTrials.gov was conducted to identify relevant studies published up to the present. Studies that examined the effects of digital health interventions, including mobile applications and web-based interventions, on perioperative care and patient outcomes in cardiac surgery were included. The data were extracted and synthesized to provide a comprehensive overview of the findings. The search yielded 15 studies composed of 4041 patients, analyzing the feasibility and implementation of mobile or internet applications for patients undergoing cardiac surgery. The studies included the use of mobile applications (ManageMySurgery, SeamlessMD, mHeart, Telediaglog, ExSed, Soulage Tavie, Heart Health application, and Mayo Clinic Health Connection) and web-based interventions (Heartnet and Active Heart). The findings indicated that these digital health interventions were associated with improved patient engagement, satisfaction, and reduced healthcare utilization. Patients reported finding the interventions helpful in their recovery process, and there was evidence of enhanced symptom monitoring and timely intervention. The completion rates of modules varied depending on the phase of care, with higher engagement observed during the acute phase. Interest in using digital health applications was expressed by patients, regardless of age, gender, or complexity of the cardiac defect. The results demonstrated that web-based interventions resulted in improvements in mental health, quality of life, and eHealth literacy. This systematic review highlights the potential benefits of digital health interventions in the context of cardiac surgery. Further research, including randomized controlled trials, is needed to establish the effectiveness, feasibility, and generalizability of digital health interventions in cardiac surgery.

## 1. Introduction

The burden of cardiovascular disease in the US is immense as it affects 80 million people [1]. Surgery is a mainstay in the treatment of many cardiovascular pathologies, including procedures, like coronary artery bypass grafting (CABG), aortic valve replacement, and heart transplant [2]. It is estimated that 313 million cardiac procedures occur globally per year; this figure will likely grow as 4.5 billion people do not currently have access to cardiac surgery [3]. Associated with these operations are massive costs as cardiac surgery accounts for 1–2% of the annual US healthcare expenditure, totaling $20 billion [4].

Though cardiac surgery is necessary in the management of many cardiovascular diseases, it can be associated with significant increases in depression and anxiety [5]. Patients who develop depression after cardiac surgery also have associated reductions in quality-of-life metrics and increased healthcare utilization [6]. A possible intervention to alleviate these problems is better patient education and engagement in the procedure itself. Patients who are well informed and involved in medical decision-making report lower rates of depression and higher quality of life [7].

Digital health is a newly burgeoning method of patient interaction. It is an easily accessible intervention as 70–80% of the world’s population owns a smartphone, and the cost of digital health applications to consumers is often negligible [8]. A common use of digital health in cardiac surgery is for post-operative screening. Telehealth visits and screening via digital health applications have been shown to be effective methods for identifying patients with post-operative complications [9,10,11]. 

In addition to its uses for provider visits and screening, digital health has the potential to be a powerful tool for education and allow patients to be active in their care [12,13]. This is an easily accessible intervention as 70–80% of the world’s population owns a smartphone [8]. A wide variety of digital health tools exist in areas, like diabetes, maternal health, and rheumatological diseases [14,15,16]. These tools are effective due to their ability to bolster self-belief by making patients active in their care and help manage patients who have complex comorbidities [12,13]. Telehealth and digital technology also have the promising potential to change diagnostic fields of medicine, such as radiology and pathology [17,18,19,20,21]. Digital technology can aid and improve the diagnostic capabilities that detect certain diseases. While digital health holds much promise, it has several limitations that prevent the realization of its full potential. 

With digital health’s novelty comes a lack of oversight. There is a struggle to keep digital health education unbiased, up-to-date, and understandable [22]. Patients with low digital health literacy, those with a low health literacy, and those with an impaired ability to navigate electronic information sources can struggle to use these interventions effectively [23]. This has led to many studies finding that digital health education is low-quality or ineffective [24]. The goal of this systematic review is to summarize the literature on digital health tools available for patients undergoing cardiac surgery and to determine their relative benefits to patients. 

## 2. Materials and Methods

### 2.1. Search Strategy

**Figure 1:**
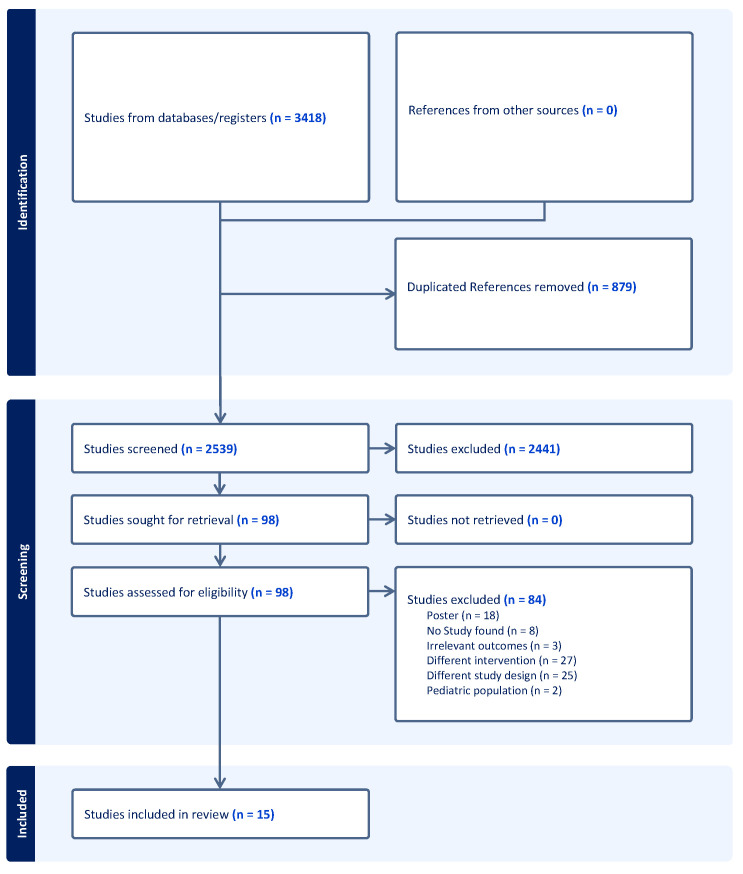
Flow diagram of study selection.

A literature search of the PubMed MEDLINE, Elsevier EMBASE, Elsevier Scopus databases (Supplementary Materials Table S1), and ClinicalTrials.gov was performed on 11 March 2022 using the keywords listed in the caption for Figure 1. PubMed MEDLINE provides comprehensive coverage of biomedical and healthcare literature, while Elsevier EMBASE specializes in biomedical and pharmaceutical research. Elsevier Scopus is a multidisciplinary database encompassing various subject areas, and ClinicalTrials.gov serves as a repository for clinical research information. This approach was designed to ensure a wide range of pertinent studies, ultimately bolstering the systematic review’s reliability and thoroughness in assessing the effects of digital health interventions on perioperative care for cardiac surgery patients. The resulting search results were put into EndNote and imported into Covidence for screening. No time limit was applied to the search as pre-determined by all investigators. After duplicated studies were removed, abstracts were screened in accordance with the Preferred Reporting Items for a Systematic Review and Meta-Analyses (PRISMA) guidelines by 2 independent authors (K.A.W. and S.K.) while applying exclusion criteria. Subsequently, pre-determined inclusion criteria were used by both independent reviewers for the evaluation of full-text studies for possible inclusion. Any disagreements were resolved by a third reviewer. 

### 2.2. Eligibility Criteria

Inclusion criteria constituted the following: any study describing the (1) development, (2) feasibility, (3) usability, and (4) clinical implementation of digital health tools or applications in patients undergoing cardiac surgery. Exclusion criteria included: (1) abstract only studies, (2) other systematic reviews, (3) pediatric studies, and (4) studies pertaining to interventions not relevant to digital health or cardiac surgery. No additional studies were identified by additional citation or hand-searching results. The flow diagram of our study selection is shown in Figure 1. Two independent authors (K.A.W. and S.K.) extracted data from the final list of included studies.

### 2.3. Risk of Bias and Quality Assessment

The assessment of bias and study quality for all included case series and cohort studies was conducted using the Methodological Index for Nonrandomized Studies (MINORS) criteria [25]. These criteria encompassed a 12-item checklist, where each criterion was assigned a score of 0 (if not reported), 1 (if inadequately reported), or 2 (if adequately reported). For noncomparative studies, the total score ranged up to 16 points, while for comparative studies, it reached up to 24 points. The quality evaluation of randomized controlled trials was accomplished using the Joanna Briggs Institute critical appraisal checklist [26]. This checklist comprises 10 items, with each item being assigned a score of “yes”, “no”, or “not reported”.

## 3. Results

### 3.1. Literature Search

The initial literature search resulted in 3418 studies. After the removal of 879 duplicate studies by both independent reviewers, the remaining 2539 studies were screened using our exclusion criteria. The remaining full texts of 84 studies were screened for eligibility using our pre-determined inclusion criteria. A total of 15 clinical studies consisting of 4041 patients were included in this study [27,28,29,30,31,32,33,34,35,36,37,38,39,40,41]. A PRISMA flowchart is illustrated in Figure 1.

The search terms used for digital health were Smartphone, Telemedicine, Telehealth, Telemonitor mHealth, eHealth, Mobile Health, Mobile, Electronic, Digital, Computer, Cellular, Online, Smart, Wearable, Device, Phone, Application, App, Portal, Telephone, Tablet, EMR, Electronic Health Record, Electronic Medical Record, Patient Portal, Internet, Web Based, Smartphone, iPad, iPhone, Android, and Smartwatch; the search included plural/singular options for all search terms.

The search terms used for cardiac surgery were Heart Transplantation, Heart Surgery, Surgery, Procedure, Operation, Valve replacement, TAVR, SAVR, Transcather Aortic Valve Replacement, and Surgical aortic valve replacement; the search included plural/singular options for all search terms.

### 3.2. Digital Health Application

Our search returned 15 studies composed of 4041 patients, analyzing the feasibility and implementation of mobile or internet applications for patients undergoing cardiac surgery (Table 1). Of those studies, two focused on heart transplantation, three focused on elective cardiac surgeries, one focused on transcatheter aortic valve replacement (TAVR), and seven focused on coronary artery revascularization either through bypass or percutaneous revascularization. All 15 studies focused on a population of patients that were at least 18 years old. Out of the 15 included studies, 13 studies focused on a mobile application and 2 studies focused on website-based interventions. The mobile applications had educational information, daily tasks, frequently asked questions, and the ability to monitor how patients were performing. The web-based interventions similarly provided educational information to patients about their concerns and post-operative management. 

### 3.3. Risk of Bias and Quality Assessment

A component of this systematic review involved assessing the potential for bias and evaluating study quality among the 15 included investigations (Table 2). The nonrandomized cohort studies achieved a minimum score of 13 out of 16 points according to the rigorous MINORS criteria, indicating a satisfactory level of methodological integrity. Similarly, the majority of the Joanna Briggs Institute criteria were met by the randomized controlled trials. This assessment helps to ensure a robust analysis of the impact of digital health interventions on perioperative care for cardiac surgery patients.

### 3.4. Randomized Control Trials

We found nine randomized control trials testing the effectiveness of digital health in perioperative care. Of these trials, seven have been completed, and two are still ongoing (Table 3).

Gomis-Pastor et al. studied the efficacy of their app in improving medication adherence for patients who had undergone heart transplant. In the study, 134 heart transplant recipients were randomized to the study intervention group or the control group who received standard care. The study app, mHeart, contains modules for education, treatment regimen, care team messaging, etc. Participant medication adherence and knowledge was measured at the baseline with SMAQ questionnaires. Participants were reassessed at follow-up, which was on average 1.6 years after the baseline. The study app group had significantly greater improvements in adherence from the baseline compared to the control group. They also exhibited greater knowledge of drug timings and indications. 

Lunde et al. studied the impact of their application on cardiovascular health for patients who recently had PCI or valve surgery. A total of 113 participants were randomized to use the study app or to receive standard care. The study app group logged their cardiovascular rehab goals into the app. The app would then provide task reminders and would act as a place where participants could enter feedback about their goals. Study supervisors who had access to this information would also provide tailored feedback to the participants as a means for motivation and education. After a year of using the intervention, the study app group had a VO_2peak_ that was 2.2 mL/kg/min greater than the control group, indicating improved cardiovascular health. The app group also had significantly improved exercise habits, exercise performance, and self-perceived goal achievement relative to the standard care group.

Martorella et al. studied the effect of the app Soulage-Tavie on pain management after cardiac surgery. A total of 60 patients scheduled for their first cardiac surgery were enrolled and randomized to the app group or standard of care group. Soulage-Tavie screened patients’ perceptions of postoperative pain and generated tailored video sessions and messages to educate patients about pain management. Data collected 7 days postop showed that while both the intervention and the control groups had similar levels of pain severity and pain catastrophizing, the app group had less pain-related interference with breathing/coughing and less pain-related barriers. 

Snoek et al. examined the effectiveness of their app in facilitating at-home cardiac rehab. A total of 179 patients with a recent history of ACS, coronary vascularization or valve intervention who declined participating in center-based cardiac rehab were enrolled. Participants were randomized to the app group or to the no intervention group. Participants in the app group followed a 6-month exercise program facilitated by the app. After 1 year, the app group had significant improvements in their VO_2peak_ relative to the baseline. Meanwhile, the no intervention group had no change in their VO_2peak_ after 1 year. 

Spindler et al. tested the viability of their app, Teledialog, as an alternative to conventional psychologic counseling. There were 136 patients with a recent history of CABG, valve surgery, ACS, or heart failure that were randomized to the Teledialog group or conventional therapy group. Teledialog is an app that allows patients to enter vitals, access educational content about living with cardiovascular disease, and facilitates e-counseling. After 12 months, both groups had equal levels of motivation for lifestyle changes, self-care, psychological distress, and quality of life. 

Widmer et al. examined their application (Personal Health Assistant) as a potential adjunct for conventional cardiac rehabilitation in patients who had PCI. Two groups of participants were recruited, those who were set to start 3 months of cardiac rehab and those who had finished cardiac rehab. Both groups were randomized to use the study app as an adjunct or to perform cardiac rehab alone, thus yielding 4 groups with 42 participants overall. The app studied acts as a log for various health tasks and offered education on methods for cardiac rehab. The group that used the application during cardiac rehab and the group that used the application after cardiac rehab had significant reductions in rehospitalization, ED visits, weight, and blood pressure relative to their respective control groups. 

Yu et al. studied the effectiveness of their application (Heart Health Application) at improving medication adherence in patients after CABG. A total of 1000 patients were randomized to the application group or standard of care group. The Heart Health Application would provide medication reminders and cardiac health education. After 6 months, there were no differences in medication adherence between the intervention and control group. There were also no differences in the rate of cardiovascular and cerebrovascular events, rehospitalization, blood pressure, body mass, or smoking status. Of note, the proportion of app usage by the intervention group decreased from 88.1% at the start of study to 9.2% at the end of the 6-month study period.

Two randomized controlled trials are still ongoing. van Steenbergen et al. developed an app aimed at reducing healthcare utilization. A total of 280 patients scheduled to undergo CABG were randomized to the app group or standard of care group. The app used would provide education on treatment, recovery, and healthy living. Outcomes of interest include healthcare utilization, anxiety, duration of recovery, quality of life, and user satisfaction. Vasankari et al. aimed to use their app ExSed to monitor and promote physical activity postop. A total of 540 patients who had undergone CABG or valve surgery were enrolled and randomized to use the app or receive conventional care alone. The ExSed app is meant to log physical activity via accelerometer data and provide users with feedback on accomplishing their activity goals. Outcomes of interest include step count, exercise capacity, quality of sleep, laboratory markers, TTE parameters, quality of life, and major cardiac events.

### 3.5. Nonrandomized Cohort Studies

We found six nonrandomized cohort studies examining the effects of using a mobile application or other digital platform as a component of their perioperative care (Table 4).

Ben-Ali et al. examined the impacts of a mobile-based application (SeamlessMD) for patients undergoing elective cardiac surgeries. In the study, 1108 patients were given the opportunity to download the mobile applications. During the four weeks before and after the surgical procedure, patients received guidance through reminders, tasks, PRO surveys, and evidence-based education. Throughout the postoperative period, patients were prompted to complete daily health surveys to monitor their recovery progress and identify any potential warning signs. The application was designed to escalate lower risk issues to self-care education and higher risk issues to the care team, such as a phone call to a nurse, based on the patient’s symptoms and signs. The application received positive feedback from patients, with 94% of them recommending it and 98% of patients finding it helpful in their recovery process. In addition, patients reported utilizing the application to prevent unnecessary health services utilization, with 45% of them avoiding at least one phone call and 28% of patients avoiding at least one hospital visit using the application.

Cook et al. tested a mobile-based application (Mayo Clinic Health Connection) on 149 patients undergoing elective cardiac surgery. The study assessed the rate at which users completed modules and found that, in total, approximately 84% of the modules were finished by the patients. The completion rate varied depending on the day, with the highest rate of 94% on the surgery day and the lowest rate of 68% on recovery Day 5.

Schuuring et al. investigated the current usage of a mobile health application (mHealth) in 118 cardiac patients. They found that there was an expressed interest in using mHealth to access information on physical health and receive guidance on how to manage symptoms or detect signs of deterioration. The analysis revealed that older age, gender, and complexity of the defect were all significantly associated with lower current smartphone usage but did not affect patients’ level of interest or preference for mHealth applications in the near future.

Venkataraman et al. studied the use of their app, ManageMySurgery (MMS), in which 69 patients underwent TAVR. Patients used the app before and after their procedure and were asked if the application was helpful in their care. A total of 73% of patients felt the application was helpful in preparation for TAVR and 86.5% would recommend that others use it before a similar procedure. After their original feasibility study, Venkataraman et al. performed a retrospective cohort study involving 388 patients with 238 patients subsequently using the MMS application after undergoing a TAVR [42]. MMS users were found to have significantly lower 90-day readmission rates, ER visits, and complication rates when compared to non-users.

Two studies examined a web-based intervention. Dew et al. used a web-based intervention (Heartnet) to look at 64 patients undergoing heart transplantation. They found patents who received the intervention experienced a decrease in depressive and anxiety symptoms, as well as a decline in anxiety and hostility symptoms among their caregivers, Furthermore, there was a significant improvement in social functioning quality of life. The mental health and quality of life benefits were more pronounced in patients who frequently used the website. Melholt et al. looked at using a web portal (Active Heart) in 109 cardiac patients. During the trial period, participants had a favorable view of the web portal, citing its ease of access, user-friendliness, and use of understandable language. Additionally, the patients’ eHealth literacy abilities improved over the course of the trial.

### 3.6. Country of Origin

The 15 studies included in this systematic review were based in countries across North America, Europe, and Asia. Four studies included were based primarily in the USA (Cook, Dew, Venkataraman, Widmer) (Table 1). Two studies were based in Canada (Ben-Ali, Martorella). Norway (Lunde, Melholt) and the Netherlands (Schuuring, van Steenbergen) each produced two studies that were included. Spain (Gomis-Pastor), Denmark (Spindler), Finland (Vasankri), and China (Yu) each contributed one study. The study by Snoek et al. was conducted across multiple countries (The Netherlands, Denmark, Switzerland, Spain, France).

### 3.7. Data Synthesis

The results from the reviewed randomized controlled trials (RCTs) and nonrandomized cohort studies demonstrate the potential of digital health interventions to transform perioperative care for patients undergoing cardiac surgery. There were improved patient engagement and medical adherence across both RCTs and cohort studies. Digital health interventions consistently demonstrated their capacity to enhance patient engagement and adherence to treatment regimens. Digital health interventions also demonstrated their effectiveness in providing tailored symptom management and symptom monitoring. In the included studies, there exists a geographic diversity, spanning North America, Europe, and Asia.

## 4. Discussion

Perioperative care is critical in managing and reducing adverse events that may arise during and after surgery [43]. Traditional perioperative care has traditionally been limited to in-person consultations, which may be resource-intensive and challenging to scale in order to meet the increasing demand for surgical procedures. Digital health interventions, such as mobile applications and web-based interventions, have emerged as potential tools to improve perioperative care, including patient engagement, self-care, and health outcomes. The COVID-19 global pandemic has accelerated the adoption of technologies [44].

### 4.1. Randomized Control Studies

The study by Snoek et al. supports that digital health can be useful in cardiac rehab. Participants who used the digital health intervention had significant improvement in cardiovascular performance. Meanwhile, the group with no intervention did not have statistically significant changes in cardiovascular performance from the baseline. These results show that digital health interventions can be a useful way to promote cardiac rehab in patients who are otherwise unable to perform traditional center-based cardiac rehab. Lunde et al. further highlight the role that digital health can play in cardiac rehab. The results show that patients using a digital health intervention had better cardiovascular fitness and exercise habits than their counterparts who used traditional center-based cardiac rehab. These data imply that digital health interventions may be able to replace center-based rehab, making post-operative care much more accessible for patients. While there is some evidence that digital health may be an alternative to center-based cardiac rehab, Widmer et al. examined the role of digital health as an adjunct. Their results show that center-based rehab with digital health resulted in lower healthcare utilization and better patient outcomes than center-based rehab alone. This is significant as digital health solutions are inexpensive and widely accessible to patients given how common smartphones are. These interventions could play a role in reducing costs to the healthcare system and ensuring patients have improved outcomes. Studies have previously showed digital health interventions can be beneficial in terms of both costs and health outcomes [45]. Patients can benefit by not having to physically go to follow-up appointments and by having increased access to healthcare. 

Aside from its use in cardiac rehab, digital health interventions show promise in promoting medication adherence. Gomis-Pastor et al. studied the utility of their digital health interventions in heart transplant recipients. Medication adherence is especially important for transplant patients given the risk of organ rejection and scarcity of transplant organs. Gomis-Pastor found their app intervention increased medication adherence as well as participants’ knowledge about medication administration and uses. This result shows that digital health can be an important tool to promote patient education leading to improved compliance. Yu et al. studied their application in patients who had undergone CABG. They did not find any significant change in medication adherence between the digital intervention group and nonintervention group. Of note, only 9.2% of participants used the app by the end of the trial period. This indicates poor adherence to the trial intervention and may explain why there were no results of significance. Further trials are needed to validate the digital intervention’s usability and patient satisfaction to ensure that patients are able to use it properly. After improving the intervention, a trial should be conducted again to see if medication adherence improves if there are high levels of digital intervention use. Digital applications have previously been used to increase medication compliance in patients with chronic medical conditions, including diabetes, hypertension, and dyslipidemia [46]. Patients are able to effectively keep track of their different medications, which becomes essential when they have to keep track of multiple medications. 

Martorella et al. studied the role of digital health in post-operative pain management. The digital health intervention reduced pain-induced barriers that participants faced despite both groups reporting similar levels of pain. These results indicate that digital health can help to improve patient quality of life by helping with the burden of symptoms’ management postop. Perhaps digital health interventions could be helpful in alleviating the burden of pain management for other surgical procedures. Post hoc analysis, however, showed that group differences were only significant on day 2. Otherwise, both groups had similar rates of opioid usage.

Spindler et al. studied the use of their app as an alternative for psychological therapy in post-op patients. It can be determined that the digital intervention was noninferior to conventional therapy. This study highlights the potential for patients to use digital health instead of conventional therapy and reap the benefits that digital health offers, such as convenience and accessibility. The value of digital health in psychological therapy needs to be further validated to show that noninferiority is not due to poorly powering the study.

### 4.2. NorRandomized Cohort Studies

The identified five nonrandomized cohort studies that examined the effects of mobile applications and web-based interventions on perioperative care suggest that digital health interventions have the potential to improve perioperative care in cardiac patients.

The study by Ben-Ali et al. found that the use of a mobile application (SeamlessMD) was associated with increased patient engagement and satisfaction. Patients found the application helpful in their recovery process and were able to prevent unnecessary healthcare utilization. Cook et al. also found that patients were engaged with the mobile application (Mayo Clinic Health Connection) during the acute phase of care, with a high completion rate of modules on the surgery day. Venkataraman et al. also found that their app (MMS) had a high level of patient satisfaction in their original feasibility study examining usage. The larger study that followed suggests that mobile applications, such as MMS, could decrease unnecessary use of emergency and inpatient care by engaging patients with a structured educational task and context [42]. The studies by Dew et al. and Melholt et al. showed that web-based interventions can be effective in improving mental health, quality of life, and eHealth literacy beyond the acute phase of the case and into the post-operative period. Their findings suggest that web-based and application interventions can complement the traditional perioperative care and enhance the patient’s recovery experience. Patients who have a higher level of compliance in the perioperative period have been shown to have superior outcomes [47,48]. These studies show that digital health interventions represent an effective way to engage and help keep patients informed during such a crucial time. 

Schuuring et al.’s study identified that patients expressed an interest in using mHealth applications to manage their symptoms and receive guidance on physical health. Interestingly, age, gender, and complexity of the defect did not affect patients’ interest in mHealth applications, suggesting that digital health interventions may be useful in reaching a diverse patient population. Despite digital platforms being fairly novel, older patients were found to have similar interest in utilizing these resources. Patients from all backgrounds may benefit from a digital health platform [49]. Previous research and studies have shown that digital platforms are typically well received although there is a learning curve associated with incorporating and implementing these interventions [50,51]. 

However, the nonrandomized cohort study design of the included studies is a limitation. It is unclear whether the observed benefits are solely attributable to the digital health interventions or whether there are other factors contributing to the results. Furthermore, the generalizability of the results is limited to the study populations and may not be applicable to other patient populations or clinical settings.

### 4.3. Country of Origin

The studies in this systematic review varied in their nation of origin with participants from the 10 countries included. The success of digital health globally showcases that it has the potential to help cardiac surgery patients regardless of geographic location; all patients require is a smartphone and access to the internet. Most of the digital interventions were based in North America or European countries, indicating the need for the development of applications and web-based interventions to help address a broader audience. With a majority of the world’s population owning a smartphone device and internet access growing, digital health interventions will only continue to become more accessible [8,52]. 

### 4.4. Limitations

There are several limitations to this systematic review. First, the study included only fully published primary studies. While this approach was adopted to ensure a methodologically stringent analysis, it does introduce the possibility of publication bias. This arises from the tendency of journals to favor the publication of studies with statistically significant results, potentially leading to an underrepresentation of studies with null or nonsignificant findings. Consequently, our review might not capture the entirety of research conducted in this domain, warranting a cautious interpretation of the cumulative evidence. Moreover, other systematic reviews were not included in the analysis, which may have resulted in the loss of in studies not captured in the review. In addition, this study excluded the pediatric cardiac population, which decreases the generalizability. Lastly, a meta-analysis was not conducted due to the differences in study design among the captured studies. Nevertheless, this systematic review offers a comprehensive overview of the existing digital health options for cardiac surgery and offers recommendations for future research and development.

## 5. Conclusions

Digital health interventions, such as mobile applications and web-based interventions, have the potential to improve perioperative care in cardiac patients. The results of the systematic review suggest that digital health interventions can enhance patient engagement, satisfaction, and health outcomes. Patients have a high level of engagement with digital health interventions, especially immediately before and after the procedure, in the peri-procedure timeline. However, further research is needed to establish the effectiveness and feasibility of these interventions in diverse patient populations and clinical settings. 

## Figures and Tables

**Table 1:** Digital Health Tools Made for Patients Undergoing Cardiac Surgery.

Study	Year	Study Design	Surgery Type	Study Population	Total Number of Enrolled or Analyzed	Intervention Name	Country
**Application**							
Venkatraman 2022 [41]	2022	Cohort study	TAVR	Adult patients undergoing TAVR	69	ManageMySurgery (MMS)	USA
Gomis-Pastor 2021 [30]	2021	RCT	Heart transplant	Post Heart Transplant	134	mHeart	Spain
Ben-Ali 2021 [27]	2021	Cohort study	elective cardiac surgery	Adult patients	1108	SeamlessMD	Canada
Cook 2014 [28]	2014	Cohort study	elective cardiac surgery	Over 50	149	MayoClinicHealthConnection	USA
Snoek 2021 [35]	2021	RCT	PCI	Patients 65 years or older who declined participation in center-based cardiac rehabilitation	179	Unnamed	5 European Countries
van Steenbergen 2021 [37]	2021	RCT	coronary artery bypass		280	Unnamed	The Netherlands
Lunde 2020 [31]	2020	RCT	N/A	Cardiac Rehab	113	Unnamed	Norway
Yu 2020 [40]	2020	RCT	CABG	18 years of age or older, had been prescribed at least one secondary preventive oral medication within 2 weeks after surgery	1000	Heart Health Application	China
Vasankari 2019 [38]	2019	RCT	coronary artery bypass, valve replacement	Adult patient doing elective surgery	540	ExSed	Finland
Spindler 2019 [36]	2019	RCT	PCI	18 years of age or above, a diagnosis of coronary artery bypass, valve surgery, heart failure or artery sclerosis, ability to understand Danish, and ability to use digital technology	136	Teledialog	Denmark
Schuuring 2016 [34]	2016	Cohort study	N/A	Congenital Heart Disease	118	Web-Based	The Netherlands
Widmer 2015 [39]	2015	RCT	PCI	Post Percutaneous Coronary Intervention (PCI)	42	personal health assistant	USA
Martorella 2012 [32]	2012	RCT	cardiac surgery requiring sternotomy	First surgery	60	Soulage Tavie	Canada
**Web-Based**							
Melholt 2018 [33]	2018	Cohort study	N/A	Patients with ischemic heart disease or CHF	49	Active Heart	Norway
Dew 2004 [29]	2004	nonrandomized trial	Heart transplant	Patients post heart transplant	64	Heartnet	USA

Abbreviations: N/A, Not Available.

**Table 2:** Risk of Bias and Quality Assessment.

**Nonrandomized Cohort Studies**									
**MINORS**	**Venkatraman 2022** [41]	**Melholt 2018** [33]	**Schuuring 2016** [34]	**Ben-Ali 2021** [27]	**Cook 2014** [28]	**Dew 2004** [29]			
A clearly stated aim	1	2	1	2	2	1			
Inclusion of consecutive patients	2	1	1	2	2	2			
Prospective collection of data	2	2	2	2	2	2			
Endpoints appropriate to the aim of the study	2	2	2	2	2	2			
Unbiased assessment of study endpoint	2	2	2	2	2	2			
Follow-up period appropriate to the aim of the study	2	2	2	2	2	2			
Loss to follow up less than 5%	0	0	2	0	0	2			
Prospective calculation of the study size	2	2	2	1	1	2			
Total	13	13	14	13	13	15			
** Randomized Control Trials **									
**Joanna Briggs Institute critical appraisal**	**Gomis-Pastor 2021** [30]	**van Steenbergen 2021** [37]	**Snoek 2021** [35]	**Lunde 2020** [31]	**Yu 2020** [40]	**Spindler 2019** [36]	**Vasankari 2019** [38]	**Widmer 2015** [39]	**Martorella 2012** [32]
Were the two groups similar and recruited from the same population?	Yes	Yes	Yes	Yes	Yes	Yes	Yes	Yes	Yes
Were the exposures measured similarly to assign people to both exposed and unexposed groups?	Yes	Yes	Yes	Yes	Yes	Yes	Not Reported	Yes	Yes
Was the exposure measured in a valid and reliable way?	Yes	Not Reported	Yes	Yes	Yes	Yes	Not Reported	Yes	Yes
Were strategies to deal with confounding factors stated?	Not Reported	Not Reported	Not Reported	Not Reported	Yes	No	Not Reported	Yes	No
Were the groups/participants free of the outcome at the start of the study (or at the moment of exposure)?	Yes	Not Reported	Yes	Yes	Yes	Yes	Not Reported	Yes	Yes
Were the outcomes measured in a valid and reliable way?	Yes	Not Reported	Yes	Yes	Yes	Yes	Not Reported	Yes	Yes
Was the follow-up time reported and sufficient to be long enough for outcomes to occur?	No	Not Reported	No	No	No	No	Not Reported	No	No
Was follow-up complete, and if not, were the reasons for the loss to follow up described and explored?	Yes	Not Reported	Yes	Yes	No	Not Reported	Not Reported	Yes	Yes
Were strategies to address incomplete follow-up utilized?	Not Reported	Not Reported	Yes	Yes	Yes	Yes	Not Reported	Yes	Yes
Was appropriate statistical analysis used?	Yes	Yes	Yes	Yes	Yes	Yes	Not Reported	Yes	Yes

**Table 3:** Randomized Controlled Trials for Digital Health Interventions.

Study	Intervention Name	Primary Objective	Secondary Objective	Primary Results	Secondary Results
Gomis-Pastor 2021 [30]	mHeart	medication adherence	medical knowledge	intervention improved med compliance	intervention increased knowledge about meds
van Steenbergen 2021 [37]	Unnamed	healthcare utilization	quality of life	reduced healthcare utilization	by improving quality of life, decreasing anxiety, and accelerating recovery
Snoek 2021 [35]	Unnamed	peak oxygen uptake (VO_2peak_) after 6 months	change in the amount of self-reported habitual physical activity was greater	peak oxygen uptake improved in the study group at 6 and 12 months	change in the amount of self-reported habitual physical activity was greater in the study group compared with the control group
Lunde 2020 [31]	unnamed	VO_2_, exercise performance	lipid panel	intervention increased VO_2_, exercise performance	intervention had no impact on lipid panel
Yu 2020 [40]	Heart Health Application	CABG secondary preventive medication adherence as measured by the MMAS-8 at the 6-month visit after randomization	secondary outcomes were mortality, major adverse cardiovascular and cerebrovascular events, cardiovascular rehospitalizations, self-reported secondary preventive medication use after 6-month follow-up, BP, BMI and self-reported smoking status	there were no significant differences in the primary outcome	there were no significant differences in the secondary clinical outcome measures
Spindler 2019 [36]	Teledialog	level of anxiety and depressive symptoms	quality of life experienced by patients	no significant differences between the two rehabilitation groups with regard to the level of anxiety and depressive symptoms	no significant differences between the two rehabilitation groups with regard to quality of life experienced by patients
Vasankari 2019 [38]	ExSed	the change in mean daily step count between the baseline (preoperatively) and at 3 months from hospital discharge	improvement in self-perceived QoL		
Widmer 2015 [39]	personal health assistant	changes in risk factors	rehospitalizations plus emergency department (ED) visits	significant reductions in weight and blood pressure	significant reductions in rehospitalizations/ED visits
Martorella 2012 [32]	Soulage Tavie	pain intensity, pain interference with daily activities, patients’ pain barriers, tendency to catastrophize in face of pain, and analgesic consumption		intervention group had less pain interference with breathing	intervention group consumed more opioid pain meds

**Table 4:** Nonrandomized Trials for Digital Health Interventions.

Study	Intervention Name	Primary Objective	Secondary Objective	Primary Results	Secondary Results
Venkatraman 2022 [41]	ManageMySurgery (MMS)	user satisfaction		73% of users found the application helpful	
Ben-Ali 2021 [27]	SeamlessMD	user satisfaction	reduction in health service utilization	94% of patients were satisfied with the app	45% of patients used the app to avoid a phone call and 28% used the app to avoid a hospital visit
Melholt 2018 [33]	Active Heart	patient satisfaction, literacy skills		patients were satisfied with app and had self-reported improved literacy	
Schuuring 2016 [34]	mHealth	use of mHealth, desire to use mHealth		only a minority of patients already used mHealth tools, but a majority would want to try	desire to use mHealth was not impacted by patients age
Cook 2014 [28]	MayoClinicHealthConnection	use by age category		age did not have a significant effect on app use	
Dew 2004 [29]	Heartnet	mental health, QoL, compliance		intervention reduced anxiety, depression and increased social QoL	

## Data Availability

No new data were created or analyzed in this study. Data sharing is not applicable to this article.

## Supplementary Materials

The following supporting information can be downloaded at: https://www.mdpi.com/article/10.3390/healthcare11172411/s1, Table S1: Search terms used for Systematic Review.
