# Supplementary material for: Digital Health for Patients Undergoing Cardiac Surgery: A Systematic Review
Source: Healthcare (Basel). 2023 Aug 28;11(17):2411. doi: 10.3390/healthcare11172411 (PMC10487407; doi:10.3390/healthcare11172411)
Supplement: Supplementary file 1 [file healthcare-11-02411-s001.zip › healthcare-2544674-supplementary.pdf]

Supplemental Table S1. Search terms used for Systematic Review

Date: 3.11.2022

Database (including vendor/platform): Ovid MEDLINE

Set #		Results
1 patients	(Patient or patients).tw.	6558410
2 Telehealth	exp Telemedicine/ or exp Internet/ or exp "mobile Applications"/ or exp "Electronic Health Records"/ or exp Smartphone/ or telemedicine.tw. or telehealth.tw. or mhealth.tw. or "tele health".tw. or "tele medicine".tw. or "m health".tw. or "e health".tw. or ehealth.tw. or "mobile health".tw. or telemonitor.tw. or telemonitoring.tw. or telemonitors.tw. or telemonitored.tw. or ((mobile or electronic or digital or computer or cellular or online or smart or wearable) adj3 (device or devices or phone or phones or applications or applications or app or apps or portal or portals or telephone or telephones or tablet or tablets)).tw. or EMR.tw. or EHR.tw. or "electronic health record".tw. or "electronic medical record".tw. or "electronic health records".tw. or "electronic medical records".tw. or EMRs.tw. or EHRs.tw. or "patient portal".tw. or "patient portals".tw. or internet.tw. or "web based".mp. or smartphone.tw. or smartphones.tw. or ipad.tw. or ipads.tw. or iphone.tw. or iphones.tw. or android.tw. or "smart watch".tw. or "smart watches".tw.	255748
3 surgery	exp "Heart transplantation"/ OR exp "Cardiac Surgical Procedures"/ OR ((heart.tw. OR hearts.tw. OR cardiac.tw.) adj4 (transplant.tw. OR transplants.tw. OR transplantation.tw. OR transplanting.tw. OR transplanted.tw.)) OR ((cardiac.tw. OR cardiovascular.tw. OR cardiothoracic.tw. OR heart.tw. OR hearts.tw.) adj4 (surgery.tw. OR surgeries.tw. OR procedure.tw. OR procedures.tw. OR operation.tw. OR operations.tw. OR operating.tw. OR operate.tw. OR operates.tw.)) OR "valve replacement".tw. OR "valve replacements".tw. OR TAVR.tw. OR SAVR.tw. OR "Transcather Aortic Valve Replacement".tw. OR "Surgical aortic valve replacement".tw. OR "Transcather Aortic Valve Replacements".tw. OR "Surgical aortic valve replacements".tw.	444,495

4 outcomes	exp "Patient Reported Outcome Measures"/ OR exp "Patient satisfaction"/ OR exp "Patient compliance"/ OR exp "Quality of Life"/ OR exp "Self care"/ OR exp Self-Management/ OR "quality of life".tw. OR compliance.tw. OR compliant.tw. OR Outcome.tw. OR outcomes.tw. OR satisfaction.tw. OR satisfactory.tw. OR satisfied.tw. OR satisfy.tw. OR satisfies.tw. OR satisfying.tw. OR "self care".tw. OR "Self management".tw. OR engagement.tw. OR engaged.tw. OR engaging.tw. OR engage.tw. OR engages.tw. OR NYHA.tw. OR KCCQ.tw. OR "Kansas City Cardiomyopathy Questionnaire".tw. OR "New York Heart Association".tw.	2,974,929
5	1 AND 2 AND 3 AND 4	443
	32812883 OR 34747896 OR 34882492 OR 32014839 OR 30079780	4/5

Database (including vendor/platform): Embase

Set #		Results
1 patients	patient:ti,ab OR patients:ti,ab	11256467
2 Telehealth	'telehealth'/exp OR 'Internet'/exp OR 'mobile application'/exp OR 'electronic health record'/exp OR 'smartphone'/exp OR telemedicine:ti,ab OR telehealth:ti,ab OR mhealth:ti,ab OR "tele health":ti,ab OR "tele medicine":ti,ab OR "m health":ti,ab OR "e health":ti,ab OR ehealth:ti,ab OR "mobile health":ti,ab OR telemonitor:ti,ab OR telemonitoring:ti,ab OR telemonitors:ti,ab OR telemonitored:ti,ab OR ((mobile OR electronic OR digital OR computer OR cellular OR online OR smart OR wearable) NEAR/3 (device OR devices OR phone OR phones OR applications OR applications OR app OR apps OR portal OR portals OR telephone OR telephones OR tablet OR tablets)):ti,ab OR EMR:ti,ab OR EHR:ti,ab OR "electronic health record":ti,ab OR "electronic medical record":ti,ab OR "electronic health records":ti,ab OR "electronic medical records":ti,ab OR EMRs:ti,ab OR EHRs:ti,ab OR "patient portal":ti,ab OR "patient portals":ti,ab OR internet:ti,ab OR "web based" OR smartphone:ti,ab OR smartphones:ti,ab OR ipad:ti,ab OR	434997

	ipads:ti,ab OR iphone:ti,ab OR iphones:ti,ab OR android:ti,ab OR "smart watch":ti,ab OR "smart watches":ti,ab	
3 surgery	'heart transplantation'/exp OR 'heart surgery'/exp OR ((heart OR hearts OR cardiac) NEAR/4 (transplant OR transplants OR transplantation OR transplanting OR transplanted)) OR ((cardiac OR cardiovascular OR cardiothoracic OR heart OR hearts) NEAR/4 (surgery OR surgeries OR procedure OR procedures OR operation OR operations OR operating OR operate OR operates)) OR "valve replacement":ti,ab OR "valve replacements":ti,ab OR TAVR:ti,ab OR SAVR:ti,ab OR "Transcather Aortic Valve Replacement":ti,ab OR "Surgical aortic valve replacement":ti,ab OR "Transcather Aortic Valve Replacements":ti,ab OR "Surgical aortic valve replacements":ti,ab	686036
4 outcomes	'patient-reported outcome'/exp OR 'patient satisfaction'/exp OR 'patient compliance'/exp OR 'quality of life'/exp OR 'self care'/exp OR "quality of life":ti,ab OR compliance:ti,ab OR compliant:ti,ab OR Outcome:ti,ab OR outcomes:ti,ab OR satisfaction:ti,ab OR satisfactory:ti,ab OR satisfied:ti,ab OR satisfy:ti,ab OR satisfies:ti,ab OR satisfying:ti,ab OR "self care":ti,ab OR "Self management":ti,ab OR engagement:ti,ab OR engaged:ti,ab OR engaging:ti,ab OR engage:ti,ab OR engages:ti,ab OR NYHA:ti,ab OR KCCQ:ti,ab OR "Kansas City Cardiomyopathy Questionnaire":ti,ab OR "New York Heart Association":ti,ab	4460835
5	1 AND 2 AND 3 AND 4	1963
6	AND [humans]/lim	1904

Database (including vendor/platform): Scopus

Set #		Results
1 patients	TITLE-ABS-KEY(Patient or patients)	9691169
2 Telehealth	TITLE-ABS-KEY(telemedicine OR telehealth OR mhealth OR "tele health" OR "tele medicine" OR "m health" OR "e health" OR ehealth OR "mobile health" OR telemonitor OR telemonitoring OR telemonitors OR telemonitored OR ((mobile OR electronic OR digital OR computer OR cellular OR online OR smart OR	1511159

	wearable) W/3 (device OR devices OR phone OR phones OR applications OR applications OR app OR apps OR portal OR portals OR telephone OR telephones OR tablet OR tablets)) OR EMR OR EHR OR "electronic health record" OR "electronic medical record" OR "electronic health records" OR "electronic medical records" OR EMRs OR EHRs OR "patient portal" OR "patient portals" OR internet OR "web based" OR smartphone OR smartphones OR ipad OR ipads OR iphone OR iphones OR android OR "smart watch" OR "smart watches")	
3 surgery	TITLE-ABS-KEY (((heart OR hearts OR cardiac) W/4 (transplant OR transplants OR transplantation OR transplanting OR transplanted)) OR ((cardiac OR cardiovascular OR cardiothoracic OR heart OR hearts) W/4 (surgery OR surgeries OR procedure OR procedures OR operation OR operations OR operating OR operate OR operates)) OR "valve replacement" OR "valve replacements" OR TAVR OR SAVR OR "Transcather Aortic Valve Replacement" OR "Surgical aortic valve replacement" OR "Transcather Aortic Valve Replacements" OR "Surgical aortic valve replacements")	301,697
4 outcomes	TITLE-ABS-KEY("quality of life" OR compliance OR compliant OR Outcome OR outcomes OR satisfaction OR satisfactory OR satisfied OR satisfy OR satisfies OR satisfying OR "self care" OR "Self management" OR engagement OR engaged OR engaging OR engage OR engages OR NYHA OR KCCQ OR "Kansas City Cardiomyopathy Questionnaire" OR "New York Heart Association")	6574702
5	1 AND 2 AND 3 AND 4	1071
